# Effects of testosterone therapy for women: a systematic review and meta-analysis protocol

**DOI:** 10.1186/s13643-019-0941-8

**Published:** 2019-01-11

**Authors:** Rakibul M. Islam, Robin J. Bell, Sally Green, Susan R. Davis

**Affiliations:** 1 Women’s Health Research Program, Monash University, Melbourne, Australia; 2 School of Public Health and Preventive Medicine, Monash University, Melbourne, Australia

**Keywords:** Testosterone, Women, Premenopause, Perimenopause, Postmenopause, Sexual function

## Abstract

**Background:**

Testosterone therapy for women is in widespread use, primarily in the form of compounded preparations and off-label use of formulations for men. The benefits and risks of such therapy remain uncertain. This review will identify and evaluate studies that have examined the effects of testosterone therapy for women on a range of outcomes including sexual function, cardiovascular events, metabolic parameters, musculoskeletal health, wellbeing, cancer events, androgenic effects and withdrawal rates.

**Methods:**

Studies meeting our pre-determined inclusion criteria will be identified through searches in Ovid MEDLINE, EMBASE, the Cochrane Central Register of Controlled Trials (CENTRAL) and Web of Science. Assessing a range of outcomes, we will assess the risk-of-bias of relevant studies and draw conclusions about the strength of evidence for benefits and risks of testosterone therapy for each outcome.

**Discussion:**

This comprehensive systematic review with meta-analysis will provide the foundation for the development of evidence-based clinical practice guidelines that will address benefits and risks of testosterone therapy, when treatment might be appropriate or inappropriate, areas of clinical uncertainty and the basis for assessment and monitoring of patients.

**Systematic review registration:**

PROSPERO registration number: CRD42018104073

## Background

Endogenous testosterone has a critical role in women’s health, either through direct androgenic action or as a consequence of conversion by the aromatase enzyme to estrogens [1]. Testosterone has important physiological roles in female reproductive and non-reproductive health. There is a physiological decline in testosterone with age that commences prior to natural menopause [2–4]. The greatest decline in circulating testosterone occurs during the late reproductive years [2]. Testosterone is often prescribed to improve sexual function in postmenopausal women presenting with low libido [5]. However, concern about the safety has been a major obstacle for the approval of testosterone therapies for women. Consequently, in most countries, testosterone therapy for women is off-label, such that the prescribed therapies are either formulations approved for men with dose modification or compounded therapy [6].

A systematic review of testosterone therapy for women indicated favourable effects on sexual function [5]. However, at the time this was published, limited safety data were available. Subsequent studies have provided further efficacy and safety data. Testosterone is administered to postmenopausal women with concurrent estrogen [7, 8] or estrogen plus progestogen therapy [9, 10] or to premenopausal women [11, 12], and postmenopausal women not using other sex steroid therapy [13]. The Endocrine Society published recommendations for the use of androgens in postmenopausal women in 2014 that included recommendations for the use of testosterone [14].

With the increasing use of compounded therapies that include testosterone, combined with the uncertainty of the role of testosterone therapy, there is a compelling need for a systematic review incorporating the more recently published clinical trials of testosterone for women that includes all modes of systemic administration studied and indications for use. This review will therefore assess the benefits and risks of using testosterone therapy for women, including effects on measures of sexual function, cardiovascular events, metabolic parameters, musculoskeletal health and wellbeing.

## Methods/design

This protocol states the conduct and reporting of a systematic review and meta-analysis in compliance with the Preferred Reporting Items for Systematic Reviews and Meta-analyses protocols (PRISMA-P) [15]. This systematic review is registered in the PROSPERO database with an identification number: CRD42018104073.

### Electronic searches

We will search the following electronic databases using Ovid software: MEDLINE, EMBASE, the Cochrane Central Register of Controlled Trials (CENTRAL) and Web of Science. These databases will be searched using the search terms shown in Table 1. The final search results will be limited to controlled clinical trials or randomised controlled trials and humans. The search strategy for Ovid MEDLINE is shown in the Appendix (Table 2).

**Table 1:** Search terms used to identify relevant studies in electronic databases

1.	menopause	7.	androgen
2.	premenopause	8.	testosterone
3.	perimenopause	9.	epitestosterone
4.	postmenopause	10.	hydroxytestosterones
5.	women	11.	methyltestosterone
6.	female	12.	testosterone propionate
13.	Population search terms: 1 OR 2 OR 3 OR 4 OR 5 OR 6		
14.	Intervention search terms: 7 OR 8 OR 9 OR 10 OR 11 OR 12		
15.	Final search terms: 13 AND 14 limited by randomised controlled trial and humans		

### Searching other resources

The reference lists of retrieved studies will be searched to identify other potentially eligible trials or ancillary publications.

#### Inclusion/exclusion of articles

We will include randomised placebo-controlled clinical trials (RCTs) published between January 1990 and July 2018. This includes crossover and parallel group designs. To be included in the review, studies will be required to (1) include women aged 18–75 years and (2) to have directly compared systemic testosterone therapy with identical placebo or a blinded comparator therapy.

Studies with non-blinded intervention/s will be excluded as the placebo for many of the outcomes of interest is high. Studies published prior to 1990 will be excluded as the effect of testosterone was not systematically investigated prior to 1990.

#### Types of interventions

We will investigate the following comparisons of intervention versus control/comparator.

##### Intervention

Systemic testosterone therapy administered as a transdermal or oral preparation, intra-muscular injection or subcutaneous implant.

##### Control/comparator

Identical appearing placebo therapy. Any concomitant therapies will be required to be the same in the intervention and comparator groups. Concomitant therapies will include estrogen alone or estrogen plus progestogen therapy.

### Types of outcome measures

#### Primary outcomes

The primary outcomes will be the effects of testosterone therapy on sexual function, cardiovascular events, metabolic parameters, cognitive function and musculoskeletal health. For example, the sexual function outcomes includes satisfying sexual events, total sexual function scores, and scores for sexual desire, arousal, orgasm, pleasure, concerns, responsiveness, sexual self-image and sexual distress.

#### Secondary outcomes

Secondary outcomes will include the effects of testosterone therapy on androgenic effects, cancer events, mood and wellbeing, and discontinuation rate. For example, the secondary outcome of androgenic effects includes acne, alopecia, clitoromegaly, increased hair growth and voice change.

### Summary of findings’ and adverse effects’ table

A ‘summary of findings table’ reporting the following outcomes listed according to priority will be presented. A ‘summary of adverse events over a 12/26/52-Week Period’ reporting the following adverse events listed according to priority will be presented.

### Data collection and analysis

#### Selection of studies

After elimination of duplicates, two review authors (RI, RB) will independently scan the abstract, title or both, of every record retrieved to determine which potential studies should be assessed further. All potentially relevant articles will be evaluated as a full text. Any discrepancy of a particular study will be resolved through discussion by the review team members. If resolving disagreement is not possible, the article will be added to those ‘awaiting assessment’ and the study authors will be contacted for clarification. The PRISMA flowchart will be used in the final review [15].

#### Data extraction and management

For studies that fulfil inclusion criteria, two review authors (RI, RB) will independently abstract key participant and intervention characteristics and report data on efficacy outcomes and adverse events. A standardised form will be developed and piloted based on the template of the Cochrane data abstraction form [16]. Any disagreements will be resolved by discussion, or if required by a third author (SD). Data will be extracted on the basic characteristics and outcomes of author/year, country/setting, study population, age, number of women randomised, intervention (placebo/treatment), duration and route of intervention, and different outcomes.

#### Dealing with duplicate publications

In case of duplicate publications or multiple reports of a primary study, all available data will be collated and the most complete dataset aggregated across all known publications will be used. In case of uncertainty, the publication reporting the longest intervention/follow-up associated with our primary or secondary outcomes will be given priority.

#### Assessment of risk of bias in included studies

Two review authors (RI, RB) will assess the risk of bias of each included study independently. We will resolve disagreements by consensus or by consultation with a third author (SD/SG).

We will assess risk of bias using the Cochrane risk of bias tool [16, 17].

We will judge ‘risk of bias criteria’ as ‘low risk’, ‘high risk’ or ‘unclear risk’ and evaluate individual bias items as described in the *Cochrane Handbook for Systematic Reviews of Interventions* [16]*.* A ‘risk of bias’ graph and a ‘risk of bias summary’ figure will be generated. The impact of individual bias domains on study results at the endpoint and study levels will be summarised. For blinding of participants and personnel (performance bias), detection bias (blinding of outcome assessors) and attrition bias (incomplete outcome data), the intention is to evaluate risk of bias separately for subjective (sexual function, wellbeing) and objective (lipid profiles, bone density) outcomes [18].

#### Measures of treatment effect

Continuous data will be expressed as standardised mean difference (CIs), while categorical data will be presented as risk ratio or absolute risk. All pooled analyses will be reported with the 95% prediction intervals accompanying the 95% confidence intervals (CIs).

#### Unit of analysis issues

We will take into account the level at which randomisation occurred, such as crossover trials and multiple observations for the same outcome.

#### Dealing with missing data

Authors will be contacted regarding missing data if feasible. Evaluation of the important numerical data such as screened, randomised participants as well as intention-to-treat and as-treated will be undertaken. We will investigate attrition rates, e.g. drop-outs, losses to follow-up and withdrawals, and critically appraise issues of missing data and imputation methods (e.g. last observation carried forward).

Where standard deviations for outcomes are not reported, we will impute these values by assuming the standard deviation of the missing outcome to be the average of the standard deviations from those studies where this information was reported. We will investigate the impact of imputation on meta-analyses by means of sensitivity analyses.

#### Assessment of heterogeneity

In the event of substantial clinical, methodological or statistical heterogeneity, we will not report study results as the pooled effect estimate in a meta-analysis. We will identify heterogeneity by visual inspection of the forest plots and by using a standard chi-square test with a significance level of *α* = 0.1, in view of the low power of this test. Heterogeneity using the *I*^2^ statistic, which quantifies inconsistency across studies, will be examined to assess the impact of heterogeneity on the meta-analysis [19]. An *I*^2^ statistic of 75% or more indicates a considerable level of inconsistency [16]. When heterogeneity is found, we will attempt to determine potential reasons for it by examining individual study and subgroup characteristics. Between-studies variation will be quantified using the DerSimonian-Laird random effects models.

#### Assessment of reporting biases

Funnel plots will be generated to assess small study effects if we include 10 studies or more that investigate a particular outcome. We will also employ Egger’s test to address the publication bias. Owing to several possible explanations for funnel plot asymmetry, we will interpret results carefully [20].

#### Data synthesis

Quantitative data will, where possible, be pooled in a statistical meta-analysis using RevMan 5.3 software. Continuous data such as the changes of sexual function score will be presented as standardised mean difference with 95% CIs. Categorical data such as the proportion of serious adverse events will be reported as a risk ratio with 95% CIs. Where statistical pooling is not possible, for example breast cancer events, the findings will be presented in narrative form where appropriate.

#### Subgroup analysis and investigation of heterogeneity

##### Sensitivity analysis

We will carry out the following subgroup analyses and plan to investigate interaction. Sensitivity analyses will be undertaken, when applicable, in order to explore the influence of the following factors on effect sizes:

- ◦ Restricting the analysis by menopausal status
- ◦ Restricting the analysis by taking into account risk of bias, as specified in the section assessment of risk of bias in included studies
- ◦ Restricting the analysis by the questionnaire validation (validated versus non-validated)
- ◦ Restricting the analysis by treatment duration (at least 12 weeks versus longer duration)
- ◦ Restricting the analysis by mode of drug delivery
- ◦ Restricting the analysis by dose of drug use
- ◦ Restricting the analysis by methods of dealing with attrition bias

We will also test the robustness of the results by repeating the analysis using different measures of effect size (such as mean difference, absolute risk, risk ratio), and our default model will be random-effects.

## Discussion

Despite the prescribing of testosterone to women which is occurring globally [21], primarily for the treatment of low libido [22], internationally agreed guidelines for the use of testosterone in women are lacking. This systematic review, with meta-analysis, will provide a comprehensive systematic analysis of clinical studies that have examined the effects of systematic testosterone therapy in women on sexual function, cardiovascular events, metabolic parameters, musculoskeletal health and wellbeing. Strengths of this review include searching all the major clinical medicine data bases and the references of retrieved articles, not limiting our search to English publications, and recontacting authors for missing data. Although we cannot fully anticipate the limitations of our review, it is possible that we will not be able to get access to all studies published in languages other than English. This review will provide a critical basis for the development of evidence-based clinical practice guidelines for the indications and contra-indications to therapy, the benefits and risks of therapy, the areas of clinical uncertainty and the assessment and monitoring of patients. Thus, it is the first step towards filling this important gap in women’s health care. The findings of this review will be presented at international conferences and published in the relevant journal/s.

## Appendix

**Table 2:** Search strategy used in Ovid MEDLINE database from 1990 to July 2018

Serial no.	Search terms	Results
1	menopause/	26,370
2	premenopause/	7244
3	perimenopause/	1121
4	postmenopause/	22,851
5	menopaus*.mp.	57,015
6	premenopaus*.mp.	19,983
7	perimenopaus*.mp.	4332
8	post?menopaus*.mp.	61,005
9	wom#n*.mp.	1,068,823
10	female.mp.	8,122,352
11	or/1–10	8,271,597
12	testosterone/	66,324
13	epitestosterone/	250
14	hydroxytestosterones/	200
15	methyltestosterone/	1409
16	testosterone propionate/	732
17	androgen*.mp.	89,237
18	testosterone.mp.	95,520
19	or/12–18	154,811
20	11 and 19	63,288
21	limit 20 to (humans and yr = “1990 -Current” and (controlled clinical trial or randomised controlled trial))	1873

## Abbreviations

CENTRAL: The Cochrane Central Register of Controlled Trials
EMBASE: International biomedical and pharmacological bibliographic database
MEDLINE: International biomedical bibliographic database
PRISMA-P: Preferred Reporting Items for Systematic Reviews and Meta-analyses Protocols
RCT: Randomised controlled trial
